# The cancer-induced lactate load and oncologic remodeling hypothesis: lactate as a driver of biosynthesis and epigenetics in cancer

**DOI:** 10.3389/fonc.2025.1638108

**Published:** 2025-10-10

**Authors:** Hüseyin Aydin

**Affiliations:** Department of Medical Biochemistry, Faculty of Medicine, Sivas Cumhuriyet University, Sivas, Türkiye

**Keywords:** CILLO hypothesis, lactate metabolism, oxaloacetate, pyruvate carboxylase, redox balance, epigenetic regulation, metabolic reprogramming

## Abstract

**Background:**

Cancer cells undergo profound metabolic reprogramming to sustain proliferation, redox homeostasis, and epigenetic remodeling. While the Warburg effect and glutaminolysis have long been recognized as central paradigms, the anabolic and regulatory role of lactate under normoxic conditions remains poorly defined.

**Hypothesis:**

The Cancer-Induced Lactate Load and Oncologic Remodeling (CILLO) hypothesis proposes that lactate, either imported through MCT1 or produced endogenously, is oxidized to pyruvate by LDHB and subsequently carboxylated to oxaloacetate (OAA) by pyruvate carboxylase. OAA then acts as a metabolic hub driving malate-dependent NADPH production, aspartate synthesis for nucleotide metabolism, activation of the serine/glycine/folate cycle, lipogenesis, and S-adenosylmethionine–mediated epigenetic modifications. In this framework, lactate is no longer a mere by-product of glycolysis but a central integrator of anabolic flux, redox balance, and chromatin dynamics.

**Conclusion:**

The CILLO hypothesis unifies previously fragmented mechanisms into a coherent paradigm, emphasizing lactate-derived carbon skeletons as active drivers of tumor growth and metabolic plasticity. Key rate-limiting steps—MCT1-mediated uptake, LDHB-dependent oxidation, PC-driven anaplerosis, and PEPCK-M–mediated cataplerosis—emerge as therapeutic nodes for intervention. This model not only advances our understanding of cancer metabolism but also suggests novel strategies for biomarker development, metabolic imaging, and targeted therapies. By reframing lactate as a central determinant of oncologic remodeling, the CILLO hypothesis provides a foundation for translational advances in oncology and personalized medicine.

## 1 Introduction

Cancer cells extensively reprogram their metabolism to ensure survival, proliferation, and fulfillment of biosynthetic demands. In this context, three major metabolic paradigms emerge: glutaminolysis, the Warburg effect, and the lactate shuttle. However, these canonical models remain insufficient to fully explain certain critical aspects of tumor metabolism, particularly the direction of carbon flux in normoxic tumor regions, compartment-specific redox regulation, and nutrient-driven mechanisms that support epigenetic programs.

Glutamine is an essential metabolite for rapidly dividing cells and contributes to nucleotide, amino acid, and lipid biosynthesis (1). Its uptake is enhanced through Solute Carrier Family 1 Member 5 (SLC1A5, ASCT2) and the sodium-dependent neutral amino acid transporters SNAT1 and SNAT2 (2). Once inside the cell, glutamine is converted to glutamate by glutaminase (GLS) and subsequently transformed into α-ketoglutarate (α-KG) via glutamate dehydrogenase (GLUD1) or aminotransferases. α-KG then enters the tricarboxylic acid (TCA) cycle, supporting both energy production and anabolic metabolism (3). Moreover, the MYC oncogene enhances GLS1 expression, thereby promoting glutamine dependence (4). GLUD1 also contributes to NADPH production, helping maintain redox homeostasis under oxidative stress conditions (5).

Glutamine additionally serves as a nitrogen donor in purine and pyrimidine biosynthesis. Thus, glutaminolysis represents a central pathway supporting energy metabolism, biosynthetic precursor generation, and oxidative defense mechanisms (6). Recent studies have shown that cancer cells exhibit metabolic flexibility by utilizing diverse energy sources such as glutaminolysis and β-oxidation (7).

The Warburg effect refers to the conversion of glucose to lactate in the cytosol despite the presence of sufficient oxygen and functional mitochondria, rather than its complete oxidation to acetyl-CoA. This metabolic shift provides three key advantages:

### a) NAD^+^ regeneration

During lactate production, cytosolic NADH is oxidized to NAD^+^, supplying the coenzyme required for glyceraldehyde-3-phosphate dehydrogenase (GAPDH) and sustaining continuous glycolytic flux.

### b) Rapid ATP production

Although glycolysis generates only two ATP per glucose molecule, the rate of ATP production is nearly ten times higher compared to oxidative phosphorylation, giving proliferating cells a metabolic advantage.

### c) Provision of biosynthetic precursors

Glycolytic intermediates serve as substrates for anabolic pathways such as nucleotide, amino acid, and lipid biosynthesis (8).

The coexistence of normoxic and hypoxic regions within the tumor microenvironment profoundly shapes cancer cell metabolic reprogramming. In addition to this environmental complexity, genetic and epigenetic alterations strongly influence the direction of metabolic pathways. For example, activation of HIF-1α and suppression of the p53 gene increase pyruvate dehydrogenase kinase (PDK) expression, leading to phosphorylation-mediated inhibition of pyruvate dehydrogenase (PDH). As a result, pyruvate is redirected toward NADH and lactate production rather than acetyl-CoA formation (9, 10).

**Figure 1:**
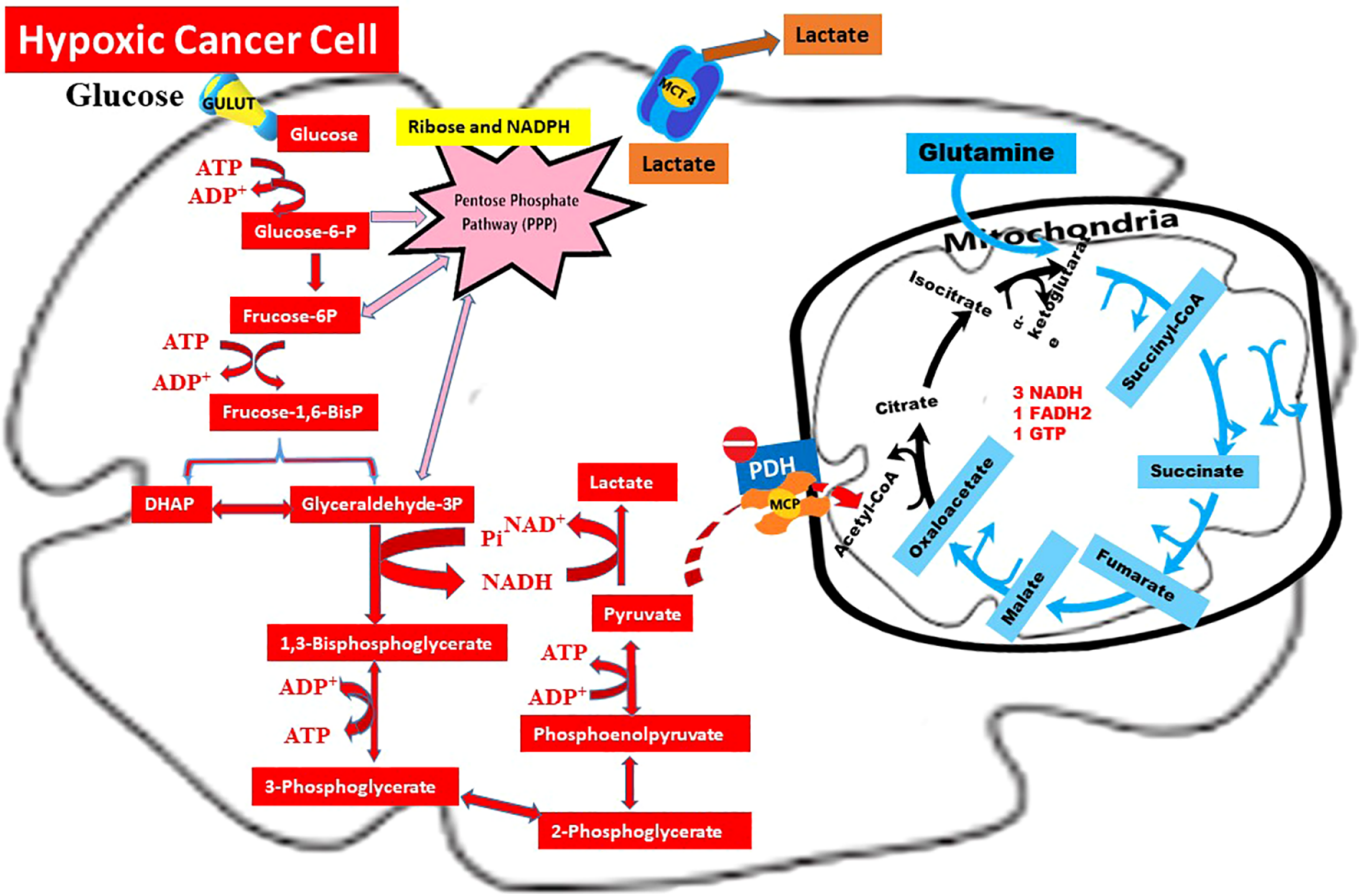
Warburg effect, glutaminolysis, and lactate shuttle in hypoxic cancer cells. Summary: This schematic illustrates the metabolic profile of a hypoxic cancer cell, where glucose is primarily metabolized into lactate through the Warburg Effect (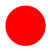). Under limited oxygen availability, pyruvate dehydrogenase (PDH) is inhibited by pyruvate dehydrogenase kinase (PDK), thereby restricting the entry of pyruvate into the mitochondrial TCA cycle. Consequently, lactate is exported via MCT4, contributing to extracellular acidification and serving as a substrate for lactate shuttling (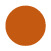) toward normoxic tumor regions. In parallel, glutaminolysis (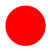) sustains anaplerotic flux into the TCA cycle through α-ketoglutarate production. Moreover, intermediates derived from glycolysis and glutaminolysis are redirected into the pentose phosphate pathway (PPP) and lipogenic pathways to generate NADPH and support macromolecule biosynthesis.

Lactate produced by hypoxic tumor cells and secreted into the extracellular matrix (ECM) is partly taken up by normoxic cancer cells, while another fraction enters the plasma and joins systemic circulation. This metabolic profile of hypoxic cancer cells, integrating the Warburg effect, glutaminolysis, and lactate shuttle, is schematically illustrated in **Figure 1**. The transfer of lactate from hypoxic to normoxic cancer cells is defined as the “lactate shuttle” and enables metabolic cooperation within the tumor microenvironment (11, 12).

## 2 Fate of lactate in rapidly proliferating cancer cells

Lactate and protons produced by hypoxic and normoxic tumor cells are transported bidirectionally across the plasma membrane by monocarboxylate transporters (MCTs), depending on the concentration gradient. Among the SLC16 gene family, MCT1 (SLC16A1), MCT2, MCT3, and MCT4 isoforms are particularly relevant (13, 14).

In hypoxic tumor cells, elevated glycolytic flux markedly increases lactate production. Under these conditions, hypoxia-inducible factor-1 alpha (HIF-1α) becomes activated and induces MCT4 expression. Although MCT4 exhibits low affinity for lactate (Km ≈ 20–35 mM), its high transport capacity allows efficient efflux of lactate and protons into the ECM. This process prevents intracellular acidosis and ensures glycolytic continuity (13, 15).

While a portion of extracellular lactate enters systemic circulation, another fraction is re-imported into normoxic cancer cells via upregulated MCT1 (Km ≈ 3–5 mM) (14, 16).

In normoxic cancer cells, enhanced glycolysis, increased lactate shuttle activity, and inhibition of the pyruvate dehydrogenase (PDH) complex collectively elevate cytoplasmic lactate levels. Intracellular lactate accumulation reverses the direction of the lactate dehydrogenase (LDH) reaction, shifting flux from pyruvate → lactate toward lactate → pyruvate. During this process, lactate is converted into pyruvate, accompanied by the reduction of NAD^+^ to NADH (17).

## 3 Fate of cytoplasmic NADH

Cytosolic NADH cannot directly cross the mitochondrial inner membrane. Therefore, it transfers its reduced electrons into mitochondria through the malate–aspartate shuttle (MAS) and glycerol-3-phosphate shuttle (G3P) systems. Electrons carried via MAS are transferred to mitochondrial NAD^+^, while the G3P shuttle mediates electron transfer through FAD. In this way, electrons derived from NADH and FADH_2_ are delivered to the electron transport chain and contribute to ATP synthesis via oxidative phosphorylation (18). These mechanisms serve to maintain cytosolic NAD^+^/NADH homeostasis.

In both shuttle systems, the regenerated cytosolic NAD^+^ supports numerous NAD^+^-dependent anabolic processes such as glycolysis, serine/glycine biosynthesis, folate metabolism, lipogenesis, and nucleotide synthesis (19). MAS is superior in terms of energy efficiency and is predominantly used in normoxic cancer cells. In contrast, the G3P shuttle functions as an alternative redox mechanism particularly in highly proliferative tumor cells. Recent literature reports that the components of the G3P shuttle are overexpressed especially in hepatocellular carcinoma, glioblastoma, and prostate cancer, and that this condition is associated with tumor progression and metabolic flexibility (20).

## 4 Fate of cytoplasmic pyruvate

In normoxic cancer cells, pyruvate arises from several sources, particularly accelerated glycolysis and the lactate shuttle. The mitochondrial pyruvate dehydrogenase complex (PDH), however, is inhibited by phosphorylation through HIF-1–induced pyruvate dehydrogenase kinases (PDKs). This inhibition restricts the conversion of pyruvate into acetyl-CoA and reduces oxidative flux, thereby contributing to cytoplasmic pyruvate accumulation (9). In a single cell, glycolysis and gluconeogenesis rarely function at full capacity at the same time. In tumors, however, spatiotemporal heterogeneity enables certain steps of gluconeogenesis, especially mitochondrial PEPCK/PCK2, to remain active. This “partial gluconeogenesis” does not culminate in glucose production. Instead, it provides biosynthetic intermediates such as phosphohydroxy acids, ribose-5-phosphate, and the glycerol backbone (21). Therefore, in cancer, gluconeogenesis is more accurately interpreted as “flux/intermediate supply–oriented” rather than “product–oriented” (22).

The principal alternative pathway for pyruvate entry into mitochondria is its anaplerotic conversion to oxaloacetate (OAA) via pyruvate carboxylase (PC); the primary role of PC is to expand the TCA intermediate pool and feed anabolic requirements such as lipogenesis through citrate export, and aspartate/thymidylate synthesis (23, 24). Clinical and experimental data demonstrate that PC expression/activity is increased in NSCLC, breast, and certain other cancers, and that PC suppression reduces proliferation, colony formation, and tumor burden (24, 25).

Alterations in glutaminolysis and fatty acid β-oxidation dynamically influence the requirement for PC. An increase in β-oxidation, accompanied by a rise in acetyl-CoA and NADH, further suppresses PDH and redirects pyruvate flux toward PC-mediated anaplerosis. In some models, glutaminolysis cannot fully compensate for the loss of PC, and dependency on PC for anaplerosis persists (26). In this context, PCK2-mediated “partial gluconeogenesis” and PC-mediated anaplerosis emerge as complementary strategies acting together not to synthesize glucose but to provide redox balance and biosynthetic precursors (27).

## 5 Metabolic fates of mitochondrial oxaloacetate

Mitochondrial oxaloacetate (OAA) cannot be directly transported out of mitochondria; therefore, it is converted into different metabolites such as citrate, malate, aspartate, or phosphoenolpyruvate (PEP), which are then transferred to the cytoplasm via specific carrier proteins.

One of the most important metabolic pathways involving mitochondrial oxaloacetate (OAA) is citrate synthesis. OAA condenses with acetyl-CoA via citrate synthase (CS). The product of this reaction is citrate. Depending on the immediate requirements of the cell, citrate may continue oxidative flux in the TCA cycle. The excess is exported from the mitochondria into the cytosol via SLC25A1—citrate carrier (CIC) located in the inner mitochondrial membrane (28, 29). In cancer, expression and/or activity of CS is frequently increased (e.g., in ovarian and prostate cancer) (30). SLC25A1/CIC levels are also elevated in many tumors, suggesting that citrate efflux from mitochondria is increased. High SLC25A1 has been associated with poor prognosis in pan-cancer analyses (31). Moreover, SLC25A1 is a transcriptional target of mutant p53 (32).

Another major fate of mitochondrial oxaloacetate (OAA) is conversion to malate. This reaction is reversibly catalyzed by malate dehydrogenase-2 (MDH2) in the mitochondrial matrix and influences TCA flux. During the reduction of OAA to malate, NADH is oxidized to NAD^+^, thereby increasing the mitochondrial NAD^+^/NADH ratio (33). The resulting malate may continue in the TCA cycle (34). The excess is exported to the cytosol via SLC25A11/OGC (malate ⇆ 2-oxoglutarate) or SLC25A10/DIC (malate ⇆ phosphate) carriers (35, 36). This axis is reprogrammed in cancer. In prostate cancer, MDH2 is elevated and associated with docetaxel resistance. In contrast, MDH2 is decreased in clear-cell renal carcinoma and affects sensitivity/ferroptosis response (37). SLC25A10 is overexpressed in many models; its suppression weakens proliferation and tumorigenesis. Germline loss-of-function variants of SLC25A11 confer hereditary predisposition to paraganglioma/pheochromocytoma (38, 39).

Another important metabolic fate of mitochondrial oxaloacetate (OAA) is its conversion to aspartate via Glutamate Oxaloacetate Transaminase 2 (GOT2). The resulting aspartate is exported from the mitochondria to the cytosol by the inner membrane carriers SLC25A12 (AGC1) and SLC25A13 (AGC2/citrin); this step is part of the malate–aspartate shuttle (MAS) and enables carbon/nitrogen flow between compartments (40).

Export of aspartate through AGC1/AGC2 allows the transfer of cytosolic→matrix NADH equivalents via MAS; under ETC inhibition/hypoxia, decreased aspartate limits proliferation (41). In addition, aspartate efflux serves cataplerotic utilization of TCA intermediates and contributes to balancing tissue-specific carbon/nitrogen fluxes (34). In the cancer context, this pathway is frequently upregulated; GOT2 and Aspartate–Glutamate Carriers (AGC1/AGC2) may enhance aspartate supply to support growth, whereas inhibition of GOT2 or blockade of aspartate transport can limit cell proliferation and tumor growth (depending on model and context) (40, 42).

Under certain cellular stress conditions or proliferative signals, mitochondrial oxaloacetate (OAA) can also be converted into phosphoenolpyruvate (PEP) and directed into the cytosol. This conversion is catalyzed by mitochondrial phosphoenolpyruvate carboxykinase-M (PEPCK-M; PCK2) in the mitochondrial matrix. During the reaction, OAA undergoes decarboxylation and phosphorylation using GTP, generating PEP and GDP (43). The resulting GDP molecules contribute to maintaining the mitochondrial GDP/GTP balance and also serve as a required cofactor for succinyl-CoA synthetase, one of the key enzymes of the TCA cycle, thereby supporting cycle continuity (44).

PEPCK-M (PCK2) expression has been shown to be significantly increased in pancreatic, lung, and colorectal tumors (43). This increase provides metabolic flexibility to cancer cells by supporting carbon flux and energy production independent of glucose. On the other hand, pharmacological or genetic inhibition of PCK2 disrupts this adaptation, reducing cell proliferation and increasing metabolic stress (45).

The identity of the carrier responsible for transporting PEP into the cytosol has not yet been fully clarified; however, recent studies suggest that this transfer occurs largely through SLC25A1 and possibly with indirect contributions from other mitochondrial carriers (46, 47). PEP reaching the cytoplasm is used not for gluconeogenesis but as a substrate for anabolic and redox-balancing pathways such as serine/glycine biosynthesis, purine/pyrimidine production, and NADPH synthesis (43, 45).

It has been shown that in lung adenocarcinomas and tumors with growth capacity under glucose-deprived conditions, mitochondrial export of PEP is accelerated, thereby directing OAA toward non-gluconeogenetic anabolic pathways (46). In this respect, cytosolic anabolic utilization of PEP derived from OAA is considered an important metabolic reprogramming strategy that reduces glucose dependence of cancer cells and supports tumor progression (43). This allows cancer cells to enhance NADPH production, redox homeostasis, and biosynthetic capacity by exploiting alternative carbon sources.

## 6 Fate of cytosolic citrate and its metabolic role in cancer

Citrate transported into the cytosol from mitochondria via the SLC25A1 carrier is cleaved by ATP-citrate lyase (ACLY) into acetyl-CoA and oxaloacetate (47). This reaction is ATP-consuming and represents an important anabolic switch upregulated in cancer (48). ACLY is highly expressed particularly in cells activated by oncogenic pathways such as PI3K/Akt/mTOR and KRAS, and this increase facilitates not only lipid synthesis but also epigenetic reprogramming processes (49).

## 7 Metabolic pathways of acetyl-CoA and its relationship with cancer

### 7.1 Fatty acid synthesis

Acetyl-CoA is converted into malonyl-CoA by acetyl-CoA carboxylase (ACC) and subsequently into long-chain fatty acids via fatty acid synthase (FASN). These lipids are used in functions such as membrane construction, signal transduction, and energy storage. In cancer, both ACC and FASN are frequently upregulated, meeting the membrane lipid demand of rapidly proliferating cells (50).

### 7.2 Cholesterol biosynthesis

Acetyl-CoA enters the mevalonate pathway via HMG-CoA synthase and subsequently HMG-CoA reductase (HMGCR) (51). This pathway enables the synthesis of cholesterol and isoprenoids. HMGCR is elevated in many cancer types, and this pathway also supports tumor progression through prenylation of oncoproteins such as Ras and Rho (52).

### 7.3 Histone/protein acetylation

Acetyl-CoA serves as a substrate for histone acetyltransferases (HATs) (53). Elevated acetyl-CoA levels increase acetylation at sites such as histone H3K9 and H3K27, thereby activating gene expression (54). This contributes to the activation of proliferative and anti-apoptotic genes in cancer cells (47).

### 7.4 Fate of cytosolic PEP

Phosphoenolpyruvate (PEP), generated in mitochondria by PEPCK-M (PCK2) and transported into the cytoplasm, classically functions as the initial substrate of gluconeogenesis (44). Cytosolic PEPCK-C (PCK1) also contributes to PEP production from OAA, serving as a key step regulating blood glucose homeostasis in hepatic gluconeogenesis (55).

In pathological conditions such as cancer, the fate of PEP, the primary substrate of gluconeogenesis, diverges from classical metabolism. It has long been known that glycolysis is markedly accelerated in cancer, and while glycolysis is active, gluconeogenic enzymes are generally suppressed. Nevertheless, studies have shown that the expression and activity of certain gluconeogenic enzymes are increased in cancer cells (43, 56). The purpose here is not glucose production; indeed, in proliferative cells, PEP provides the carbon backbone for phospholipid and serine/glycine biosynthesis, thereby indirectly contributing to nucleotide synthesis and methylation cycles (45).

Cytosolic PEP participating in the gluconeogenic pathway is first converted into 2-phosphoglycerate and 3-phosphoglycerate via enolase and phosphoglycerate mutase, respectively; then, 1,3-bisphosphoglycerate is formed by phosphoglycerate kinase. This intermediate is reduced to glyceraldehyde-3-phosphate (GAP) by glyceraldehyde-3-phosphate dehydrogenase (GAPDH), directly influencing the NADH/NAD^+^ balance. GAPDH is frequently overexpressed in cancer cells and contributes to tumor cell survival not only through energy production but also through non-metabolic functions such as nuclear localization and gene regulation (57). Thus, the steps from PEP to GAP play a critical role in balancing the increased redox demand in cancer and in supplying biosynthetic intermediates.

The GAP obtained is converted into dihydroxyacetone phosphate (DHAP) by triose phosphate isomerase (TPI). Increased TPI expression and its reprogramming through post-translational modifications (phosphorylation, nitrosylation) in cancer cells shift the GAP ↔ DHAP balance toward lipid biosynthesis (58). DHAP is reduced to glycerol-3-phosphate by glycerol-3-phosphate dehydrogenase (G3PDH) using NADH, and this metabolite forms the backbone of phosphatidic acid and subsequently phospholipid synthesis. During this reaction, NAD^+^ is regenerated, contributing to NAD^+^/NADH homeostasis. This pathway provides a critical adaptation in rapidly proliferating cancer cells to meet the increased demand for membrane phospholipids and to maintain redox balance (50).

In the gluconeogenic pathway, PEP proceeds through successive reactions up to 3-phosphoglycerate (3-PG). At this point, 3-PG is oxidized to 3-phosphohydroxypyruvate via phosphoglycerate dehydrogenase (PHGDH) using NAD^+^. Subsequently, phosphoserine aminotransferase (PSAT1) transfers an amino group from glutamate to this intermediate, synthesizing 3-phosphoserine; in the final step, phosphoserine phosphatase (PSPH) produces serine (59). Serine not only participates in protein synthesis but also serves as the main source of one-carbon units and NADPH production via the folate cycle (60).

Glycine derived from serine acts as an important substrate for nucleotide biosynthesis, participating in both purine and pyrimidine synthesis (61). Therefore, the flux starting from PEP and directed toward serine and glycine through 3-PG provides essential building blocks for DNA, RNA, and methylation reactions in the cell.

From a cancer biology perspective, the serine/glycine pathway is of great significance. Amplification and overexpression of PHGDH have been reported in many tumor types, particularly melanoma, lung, and breast cancers (59). Increased PHGDH enables glycolytic carbons to be diverted into serine/glycine biosynthesis, thereby enhancing the nucleotide and redox capacity required for cell division. Indeed, inhibition of this pathway reduces proliferation and increases the sensitivity of cells to metabolic stress (61).

## 8 Fate of cytosolic malate

Malate exported to the cytosol is reconverted into oxaloacetate (OAA) and NADH by cytosolic malate dehydrogenase (MDH1) using NAD^+^. The resulting cytosolic OAA is converted to phosphoenolpyruvate (PEP) by PEPCK-C and enters numerous metabolic pathways.

In cancer: Expression and activity of MDH1 are frequently elevated. For example, in pancreatic and lung cancers, overexpression of MDH1 facilitates the conversion of malate to OAA, thereby enhancing PEP production and anabolic metabolism (62). This increase not only directs carbon flux toward serine/glycine biosynthesis and nucleotide synthesis via PEP but also supports cytosolic redox homeostasis through NADH production. NADH can be directed toward lactate production via lactate dehydrogenase (LDH), maintaining the NAD^+^/NADH balance and contributing to the energy requirements of tumor cells with high glycolytic rates (37). Excessive MDH1 activity has also been shown to increase proliferation in cancer and strengthen cellular resistance to metabolic stress (63). Therefore, MDH1 is considered a nodal point with both biosynthetic and redox-regulatory functions in certain tumor types.

Malate transported into the cytosol is converted by the NADP^+^-dependent malic enzyme (ME1) through oxidative decarboxylation into pyruvate + CO_2_ + NADPH; this reaction directly feeds the cytosolic NADPH pool (64).

NADPH produced by ME1 is used by Fatty Acid Synthase (FASN) in fatty acid synthesis and as an essential electron donor in antioxidant defense via the glutathione and thioredoxin systems; thus, ME1 flux supports both lipogenesis and oxidative stress tolerance (65).

This flux generally follows the pathway citrate → ACLY → OAA → (MDH1) → malate → (ME1) → pyruvate + NADPH; pyruvate can then re-enter the mitochondria to be directed toward acetyl-CoA by PDH or OAA by PC, thereby ensuring integration between cytoplasmic and mitochondrial carbon and reducing equivalents (47).

In cancer, particularly in KRAS-dependent pancreatic adenocarcinoma, the Glutamate Oxaloacetate Transaminase 1 (GOT1)-MDH1-ME1 axis is reprogrammed to enhance NADPH production; silencing GOT1 or suppressing ME1 leads to intracellular ROS accumulation, redox imbalance, and loss of proliferation (66).

NADPH supplied by ME1, together with NADPH derived from the pentose phosphate pathway (G6PD) and Isocitrate Dehydrogenase 1 (IDH1), forms complementary reservoirs for lipid biosynthesis and redox homeostasis in tumors; their relative contributions vary depending on cell type and oncogenic drivers (65).

Aspartate transported into the cytosol via AGC1/2 carriers participates in various metabolic pathways. The reconversion of aspartate into OAA in the cytosol is catalyzed by AST/GOT1; this process has been discussed in detail above under the section on OAA metabolism (55). Beyond this, aspartate is a critical metabolite particularly for nucleotide biosynthesis and nitrogen metabolism.

Aspartate is an essential substrate for both purine and pyrimidine biosynthesis. In purine biosynthesis, aspartate contributes as a nitrogen donor to the purine ring during the later stages of inosine monophosphate (IMP) synthesis. In pyrimidine synthesis, it combines with carbamoyl phosphate to initiate the orotate pathway, contributing to uridine monophosphate (UMP) formation (61). Therefore, in proliferative cells, aspartate availability is considered a limiting factor for DNA/RNA synthesis. Indeed, under conditions of electron transport chain inhibition, mitochondrial aspartate production decreases, cytosolic aspartate levels fall, and tumor cell proliferation ceases (41).

Another important fate of aspartate is its involvement in the urea cycle. In the cytosol, argininosuccinate synthase (ASS1) combines aspartate with citrulline to form argininosuccinate. This step is critical for the detoxification of nitrogen as urea in cancer cells with accelerated amino acid catabolism. However, in many tumors, ASS1 expression is suppressed. This results in the diversion of aspartate away from the urea cycle and toward nucleotide biosynthesis, thereby enhancing the proliferative capacity of the tumor (67). Thus, aspartate is under dual metabolic pressure: tolerating the nitrogen load imposed by increased amino acid catabolism on the one hand, and sustaining DNA/RNA synthesis on the other.

In conclusion, aspartate exported into the cytosol is not limited to conversion into OAA. Through nucleotide biosynthesis, the urea cycle, and the malate–aspartate shuttle, it provides both the building blocks necessary for cell division and contributes to the maintenance of nitrogen and redox balance. In cancer, accelerated amino acid catabolism further increases aspartate demand by accelerating urea metabolism. Therefore, aspartate production and transport represent a limiting factor for proliferation and a central node in the reprogramming of cancer metabolism (66).

## 9 CILLO hypothesis

The Cancer-Induced Lactate Load and Oncologic Remodeling (CILLO) Hypothesis offers a new and original perspective on cancer metabolism. According to this hypothesis, the fate of pyruvate is not limited solely to its reduction to lactate. Lactate is not merely an energy substrate, an immune-evasion tool, or a waste product; rather, it functions as a critical metabolite in biosynthetic and redox processes, as well as in mechanisms that shape tumor prognosis.

The Warburg effect, glutaminolysis, and the lactate shuttle have largely been addressed separately in the literature. The CILLO Hypothesis not only integrates these three axes within a common framework. It also provides an expanded perspective that includes additional metabolic orientations such as pyruvate carboxylase–based anaplerosis, NADPH-producing pathways, and the serine/glycine–folate cycle. Furthermore, it incorporates epigenetic acetylation and the biosynthesis of phospholipids, fatty acids, cholesterol, and isoprenoids via citrate–acetyl-CoA. In this way, it proposes a comprehensive model that explains the metabolic flexibility unique to cancer cells.

## 10 In this model

In hypoxic cells, the reduction of glucose to lactate ensures NAD^+^ regeneration. In normoxic cells, lactate is converted to pyruvate and then to oxaloacetate (OAA) via pyruvate carboxylase. OAA is directed to different biosynthetic and redox pathways:

### 10.1 Citrate cycle

OAA combines with acetyl-CoA to form citrate. When citrate is transported to the cytosol, it is reconverted to acetyl-CoA by ATP-citrate lyase (ACLY). This acetyl-CoA is used in lipid and sterol biosynthesis and also contributes to epigenetic regulation through histone and DNA acetylation.

### 10.2 Malate cycle

OAA is converted to malate via MDH1 while producing NADH. When malate is converted to pyruvate via ME1, it provides NADPH. NADPH is critical for lipogenesis and antioxidant defense. In particular, in KRAS-mutant pancreatic cancer, the GOT1–MDH1–ME1 axis has been shown to be reprogrammed to increase NADPH production.

### 10.3 Aspartate metabolism

Aspartate is essential for nucleotide biosynthesis. Although aspartate can normally participate in the urea cycle, in many tumors it is preferentially directed toward nucleotide synthesis via suppression of ASS1. Therefore, aspartate supply is a limiting factor for proliferation. Under conditions where the electron transport chain is inhibited, reduced mitochondrial aspartate production halts proliferation.

### 10.4 PEP pathway

Phosphoenolpyruvate (PEP) derived from OAA proceeds to 3-phosphoglycerate (3-PG) and bifurcates into two branches: (i) 3-PG → DHAP → glycerol-3-phosphate → phospholipid biosynthesis, (ii) 3-PG → serine/glycine biosynthesis → folate cycle → nucleotide synthesis and NADPH production. Thus, PEP becomes a central metabolite for both membrane biogenesis and redox homeostasis.

### 10.5 Rate-limiting steps

Within the CILLO hypothesis, four enzymatic steps emerge as potential rate-limiting nodes: (i) MCT1-mediated lactate uptake, (ii) LDHB-mediated lactate → pyruvate conversion, (iii) PC-mediated pyruvate → oxaloacetate anaplerosis, and (iv) PCK2-mediated OAA → PEP cataplerosis. Each of these steps should be considered critical checkpoints that can limit OAA-derived biosynthetic pathways (serine/glycine, aspartate, citrate) and NADPH/NAD^+^ regeneration in the tumor cell.

The originality of the CILLO Hypothesis lies in explaining how these metabolites, through gluconeogenesis-based yet non–glucose-producing orientations, serve the cancer cell’s increased growth capacity, redox balance control, and microenvironmental adaptation.

**Figure 2:**
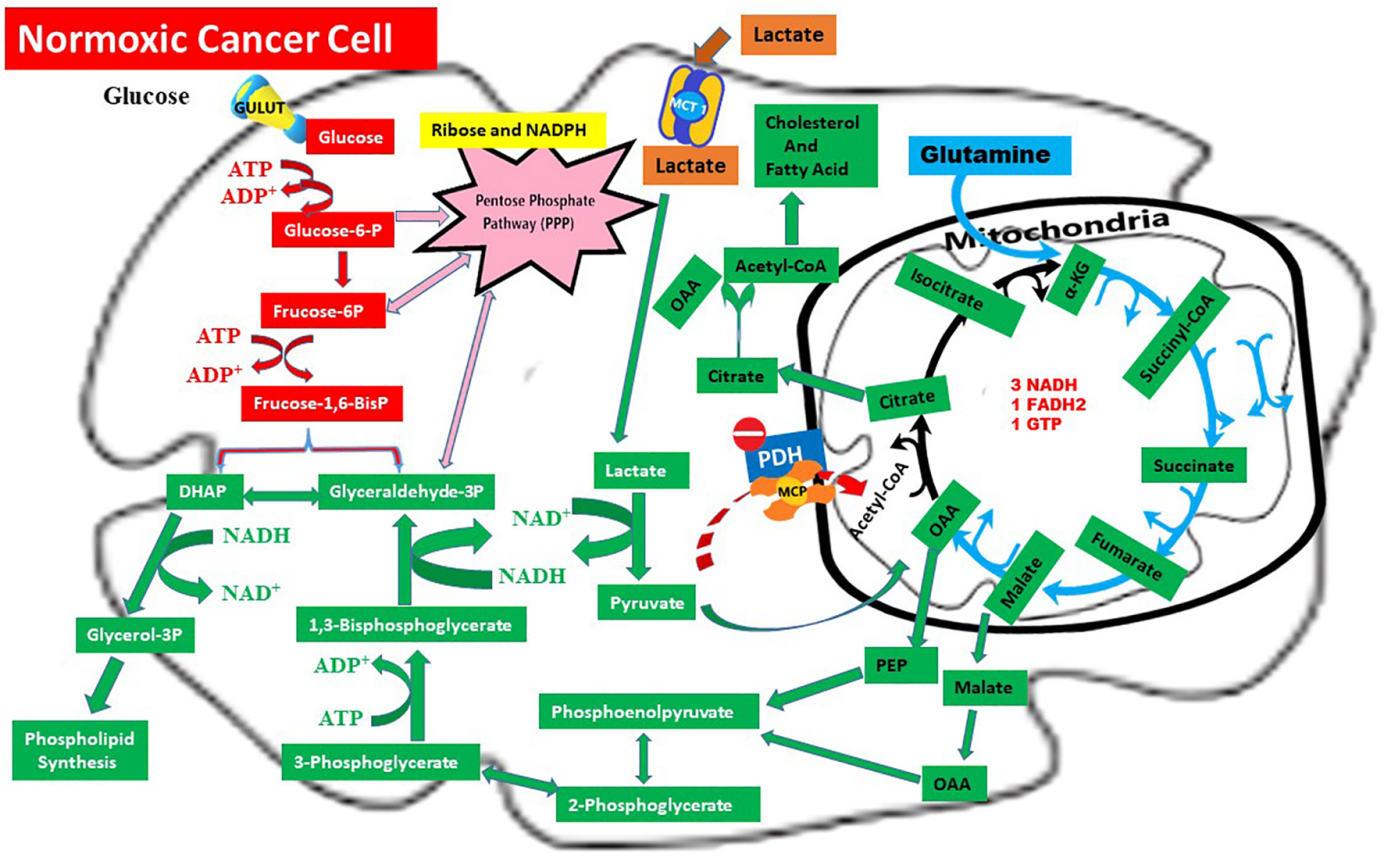
Metabolic rewiring in normoxic cancer cells: The CILLO hypothesis. 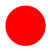 Red arrows: Warburg Effect, 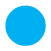 Blue arrows: Glutaminolysis, 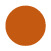 Brown arrows: Lactate Shuttle, 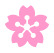 Pink arrows: Pentose Phosphate Pathway (PPP), 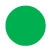 Green arrows: CILLO Hypothesis.

In conclusion, the CILLO Hypothesis organizes mechanisms dispersed in the literature into a coherent structure; it explains cancer’s unique metabolic adaptations at the systems level and lays the groundwork for identifying new therapeutic targets for metabolic intervention strategies. Lactate flux in the normoxic cell and details of the CILLO hypothesis are shown in **Figure 2**.

## 11 Contribution of the CILLO hypothesis to the literature and its scientific significance

The CILLO Hypothesis presents a new paradigm that goes beyond long-described metabolic processes in the field of cancer biology by uniting these mechanisms within a holistic network framework. Although pathways such as the Warburg effect, glutaminolysis, and the lactate shuttle have been studied in detail for decades, with comprehensive molecular mechanisms proposed for each, these studies have mostly focused on individual metabolites, specific cell types, or isolated pathways. Thus, although the literature is rich, it is fragmented and dispersed.

The contribution of the CILLO Hypothesis is to integrate this fragmented information at the systems level. It interprets the metabolic orientations of cancer cells along the lactate–pyruvate–OAA axis not only in terms of energy production, but also within the broader context of anabolic growth, redox homeostasis, nucleotide synthesis, and microenvironmental adaptation.

We can gather the original contributions of this hypothesis to the literature under the following headings:

### a) Metabolic integration approach

The CILLO hypothesis provides an integrated framework explaining how key intermediates transported between mitochondria and cytosol (particularly lactate, pyruvate, and OAA) are utilized in both classical energy pathways and alternative biosynthetic orientations. Thus, unlike existing hypotheses, it focuses not only on a single metabolite or pathway but on the reciprocal flux between them and on metabolic flexibility.

### b) A new perspective on the fragmented use of gluconeogenesis

It presents, in the context of cancer, alternative routes in which gluconeogenesis steps (e.g., PEP → 2,3-BPG or glycerol-3-phosphate) are used for anabolic and functional orientations without culminating in classical glucose synthesis. These orientations are linked to growth, membrane synthesis, and oxygen transport dynamics in tumor cells.

### c) A multidimensional interpretation of metabolic flexibility

The CILLO hypothesis defines not only the substrate diversity of cancer cells but also the roles of metabolites in modulating the extracellular microenvironment, their contributions to intracellular signaling, and adaptive strategies that simultaneously manage the energy–carbon balance.

### d) Contribution to the identification of new therapeutic targets

The CILLO hypothesis proposes evaluating enzymes such as MCT1, LDHB, PC, ACLY, and BPGM not only through their biochemical functions but also as key control points of metabolic flexibility.

In sum, the CILLO Hypothesis is not merely a summary of existing knowledge; rather, it reorganizes this knowledge with a new scheme of organization and in order of functional priority. Thus, it brings a systemic and dynamic understanding to cancer metabolism. Just as Warburg’s definition of aerobic glycolysis was a turning point as a hallmark of cancer, the CILLO hypothesis reinterprets cancer’s metabolic identity by emphasizing the central role of the lactate–pyruvate–OAA axis.

## 12 Organ-specific metabolic differences

Different organ tumors utilize lactate metabolism in unique contexts. In glioblastoma, lactate contributes to immune evasion by suppressing CD8^+^ T-cell functions and by creating an immunosuppressive microenvironment through histone lactylation. In the liver, “glucose-non-producing gluconeogenesis” via PCK2 supplies carbon to serine/glycine and nucleotide biosynthesis and has been associated with proliferation and poor prognosis in hepatocellular carcinoma. In lung and breast cancers, overexpression of PC is decisive in meeting the needs for lipogenesis and anaplerosis by directing pyruvate to OAA. These organ-specific variations indicate that the CILLO hypothesis may have heterogeneous clinical implications across different tumor types.

## 13 Contribution of the CILLO hypothesis to therapeutic approaches

The CILLO Hypothesis opens new horizons in the development of therapeutic strategies by revealing that the metabolic orientations executed by cancer cells along the lactate, pyruvate, and OAA axis are not only biological but also clinically targetable processes. The holistic metabolic model offered by this hypothesis provides a foundation for going beyond current treatments to break cancer’s metabolic flexibility, limit tumor adaptation, and target specific biosynthetic needs.

Combining metabolic targets with immunotherapy and classical chemotherapies increases the clinical relevance of the CILLO axis. For example, MCT1 inhibition with AZD3965, when considered together with programmed cell death protein 1 blockade, may reduce immune suppression; PC and PCK2 inhibition may show synergy with antifolate therapies; and BPGM modulation may regulate erythrocyte oxygen delivery and disrupt hypoxia adaptation. These combination strategies offer a new framework for personalized treatments following tumor type–specific metabolic profiling.

### 13.1 Disrupting metabolic adaptation

The CILLO hypothesis shows that under different microenvironmental conditions (e.g., hypoxia, glutamine limitation, redox stress), cancer cells activate alternative routes through lactate, pyruvate, and OAA. These adaptive metabolic networks contribute to the survival of cell subpopulations resistant to classical chemotherapy. Therefore, targeting the strategic nodes of these pathways allows the weakening of therapy-resistant tumor cells.

### 13.2 New target molecules

Within the framework of the CILLO Hypothesis, enzymes and transporters such as monocarboxylate transporter 1 (MCT1), pyruvate carboxylase (PC), and glycerol-3-phosphate dehydrogenase (GPD1) emerge as potential therapeutic targets. MCT1, which enables the use of lactate for mitochondrial oxidation and anabolic processes in normoxic cells, can be targeted particularly with inhibitors such as AZD3965. In this way, by preventing lactate uptake into the cell, metabolic competition is disrupted and oxidative tumor cells can be suppressed. PC plays a critical role in maintaining anaplerosis via oxaloacetate production and citrate-centered lipid biosynthesis; therefore, PC inhibitors may interrupt the TCA cycle and related metabolic pathways, thereby limiting cellular proliferation. GPD1 contributes to NAD^+^ regeneration and phospholipid synthesis via glycerol-3-phosphate. Targeting this enzyme may both disturb redox balance, straining cellular homeostasis, and restrict membrane lipid biosynthesis, weakening the adaptive capacity of tumor cells.

### 13.3 Disrupting redox homeostasis

Metabolic nodes that balance NAD^+^/NADH and NADP^+^/NADPH through enzymes such as GAPDH, GPD1, ME1, and IDH1 play a critical role within CILLO. By targeting these redox centers, the tumor cell’s resistance to oxidative stress can be broken. Especially in combination therapies, these targets may act synergistically with drugs that increase DNA damage.

### 13.4 Suppressing anabolic pathways

As the hypothesis posits, the glycerol-3-phosphate, serine/glycine, and aspartate routes derived from PEP support the synthesis of nucleotides, phospholipids, and amino acids in tumors. Inhibiting these pathways can directly affect the cell’s proliferative capacity. The use of inhibitors targeting these routes may be meaningful particularly in tumors with high proliferative potential.

### 13.5 Disrupting metabolic interactions with the tumor microenvironment

Lactate is known to create an immunosuppressive microenvironment and to establish synergistic relationships with stromal cells. MCT inhibitors or blockade of lactate production/uptake pathways can break these interactions and increase sensitivity to immunotherapies.

The CILLO Hypothesis is not only a metabolic explanation but also a framework that paves the way for multilayered therapeutic intervention strategies. Going beyond the literature that relates the Warburg effect to energy production, glutaminolysis to nitrogen and energy metabolism, and the lactate shuttle to intercellular symbiotic metabolic interaction, it defines the simultaneous utilization, interdependence, and adaptive reorganization of these pathways. Thus, the CILLO hypothesis offers a new basis that can contribute to more effective targeting of therapy-resistant cancer subtypes, the development of combined metabolic intervention strategies, and personalized treatment approaches.

An acidic microenvironment suppresses T-cell functions and increases the risk of immune resistance via the PD-1 (programmed cell death protein 1)/PD-L1 (programmed death-ligand 1) axis. Therefore, combining MCT1 inhibitors with immune checkpoint inhibitors may be a promising strategy for reprogramming the tumor microenvironment.

## 14 Biomarker development potential of the CILLO hypothesis

By proposing that cancer cells use lactate, pyruvate, and OAA metabolism as an integrated network, the CILLO Hypothesis enables the evaluation of metabolites and associated enzymes in this axis as biomarkers. In particular, the fact that these molecules are directly related to the tumor cell’s capacity for metabolic adaptation suggests that biochemical indicators based on the CILLO hypothesis may provide important clinical signals for diagnosis, prognosis, and treatment response. Findings that appear in a fragmented fashion in the literature can be placed into a systematic framework with this hypothesis, allowing the potential biomarkers to be evaluated in a logical context. In this scope, possible biomarkers that may be observed in cancerous tissue or in circulating biological fluids include plasma lactate/pyruvate ratio, MCT1/MCT4 expression ratio, pyruvate carboxylase (PC) activity, glycerol-3-phosphate dehydrogenase (GPD1) expression, and citrate/malate/aspartate levels. Each of these markers can provide information about the tumor cell’s substrate utilization, mitochondrial/cytosolic fluidity, oxygenation status, and biosynthetic orientation. Furthermore, when these parameters are made monitorable by non-invasive methods such as liquid biopsy, they may become valuable tools for early diagnosis in cancer, monitoring of treatment response, or evaluating the efficacy of metabolically targeted therapies.

The clinical validity of biomarkers based on the CILLO hypothesis has been demonstrated in studies conducted in recent years. In glioblastoma, the lactate–immune signature shows potential for predicting response to immunotherapy. In hepatocellular carcinoma, PCK2 levels have been associated with proliferation and prognosis. In breast and lung cancers, PC expression correlates with tumor burden and treatment resistance. These findings indicate that biomarkers within the CILLO axis may have tumor type–specific diagnostic and prognostic value.

## 15 Methods for experimental validation of the CILLO hypothesis

Multilayered experimental approaches are required to validate the metabolic orientations and anabolic/redox contributions along the lactate–pyruvate–OAA axis proposed by the CILLO Hypothesis. In this regard, in cell culture models under different oxygen concentrations and substrate limitations, the metabolic flexibility of cancer cells can be tested. Tracking metabolites such as glucose, glutamine, and lactate with labeled isotopes (e.g., ¹³C or ¹^5^N) enables quantitative monitoring of carbon flux directions through LC–MS/MS–based stable isotope tracing (SIRM) analyses.

At the same time, expression levels and activities of critical enzymes such as ATP-citrate lyase (ACLY), pyruvate carboxylase (PC), phosphoenolpyruvate carboxykinase (PEPCK), GAPDH, ME1, LDHB, MCT1, and SLC25A1 can be analyzed by qRT-PCR (gene level), Western blot and ELISA (protein level), and immunoprecipitation assays. Enzyme activity measurements can be performed with colorimetric or fluorometric spectrophotometric assays that track NADH/NAD^+^ or NADP^+^/NADPH production.

In addition, outputs such as intracellular redox status (NADPH/NADP^+^, GSH/GSSG ratio), cell division rate, mitochondrial function, lipid synthesis, and epigenetic modifications (e.g., histone acetylation, lactylation) can also be measured quantitatively under cell culture conditions, enabling the hypothesis to be tested at the systems level.

## 16 Conclusion

The CILLO Hypothesis posits that cancer cell metabolism is not merely a system focused on energy production, but rather an adaptable and versatile network that is highly responsive to environmental conditions. This hypothesis reveals that the metabolic orientations shaped around the lactate–pyruvate–oxaloacetate (OAA) axis cannot be evaluated solely within the framework of the classical Warburg effect or glutaminolysis; instead, these metabolites play multilayered roles in epigenetic regulation, redox balance, and anabolic synthesis pathways.

In the current literature, it is widely accepted that lactate is active not only in energy metabolism but also in processes such as immune suppression, reprogramming of stromal cells, and epigenetic modifications (e.g., histone lactylation). Similarly, overexpression of pyruvate carboxylase in certain cancer types and the production of OAA have been shown to mediate gluconeogenesis-like anabolic fluxes by directing NAD^+^ regeneration, aspartate biosynthesis, and serine/glycine/phospholipid metabolism (68). By addressing these metabolic orientations not in a fragmented manner but through a holistic approach, the CILLO Hypothesis explains the reprogramming of the cancer cell at the systems level.

The originality of the hypothesis lies in proposing that lactate contributes to energy production by being transported from hypoxic to normoxic regions. In addition, in normoxic cancer cells, lactate participates in gluconeogenesis-like reactions through the production of mitochondrial OAA and phosphoenolpyruvate (PEP). These processes support the generation of intermediate metabolites. These orientations both ensure the sustainability of glycolytic flux by providing NAD^+^ regeneration and maintain redox homeostasis to establish a biochemical substrate favorable for proliferation.

The CILLO Hypothesis also reveals that this metabolic network offers potential therapeutic targets (e.g., MCT1, PC, GAPDH, ME1) and biomarker candidates (e.g., cytosolic PEP, OAA, NADPH/NADP^+^ ratio). Thus, it may contribute not only to a better understanding of cancer but also to the development of personalized treatment approaches.

In conclusion, the CILLO Hypothesis offers a new paradigm based on the lactate–pyruvate–OAA axis that integrates mechanisms dispersed in the existing metabolic literature. This holistic approach shows that cancer metabolism is not solely focused on energy production but is also reorganized to serve biosynthetic and redox needs. The hypothesis provides an important framework that can lead to new research in both basic science and translational oncology.

## 17 Summary

This figure depicts the integrated metabolic adaptations of a normoxic cancer cell, highlighting the central role of lactate in biosynthetic pathways through the CILLO Hypothesis (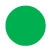). Lactate imported via MCT1 (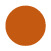) is converted to pyruvate by LDHB. Instead of fueling oxidative metabolism through PDH, pyruvate is redirected to oxaloacetate (OAA) by mitochondrial pyruvate carboxylase (PC). OAA and its derivatives support multiple anabolic processes: malate can generate cytosolic NADPH via malic enzyme (ME1), citrate export and ATP-citrate lyase (ACLY) fuel lipid and cholesterol synthesis, and phosphoenolpyruvate (PEP) links to serine/glycine and folate metabolism as well as aspartate-derived nucleotide synthesis.

Meanwhile, glucose metabolism proceeds through glycolysis and the Warburg pathway (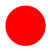), with diversion into the pentose phosphate pathway (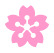) to generate ribose-5-phosphate and NADPH for nucleotide biosynthesis and redox balance. In parallel, glutaminolysis (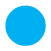) sustains TCA cycle anaplerosis via α-ketoglutarate, ensuring metabolic flexibility.

## Data Availability

The original contributions presented in the study are included in the article/supplementary material. Further inquiries can be directed to the corresponding author.
